# Supplementary material for: TBM Hunter: Identify and Score Canonical, Extended, and Unconventional Tankyrase-Binding Motifs in Any Protein
Source: Int J Mol Sci. 2023 Nov 30;24(23):16964. doi: 10.3390/ijms242316964 (PMC10706912; doi:10.3390/ijms242316964)
Supplement: Supplementary file 1 [file ijms-24-16964-s001.zip › ijms-2710084-supplementary.pdf]

Supporting Information

TBM Hunter: identify and score canonical, extended, and unconventional tankyrase-binding motifs in any protein

Christopher M. Clements [1], Samantha X. Shellman [2], Melody H. Shellman [3] and Yiqun G. Shellman [1,4,*]

Department of Dermatology, School of Medicine, University of Colorado Anschutz Medical Campus, Aurora, CO 80045, USA; christopher.m.clements@cuanschutz.edu

2 Department of Computer Science, University of Colorado Boulder, Boulder, CO 80309, USA; samantha.shellman@colorado.edu

3 H. Milton Stewart School of Industrial and Systems Engineering, Georgia Institute of Technology, Atlanta, GA 30332, USA; mshellman3@gatech.edu

4 Charles C. Gates Regenerative Medicine and Stem Cell Biology Institute, School of Medicine, University of Colorado Anschutz Medical Campus, Aurora, CO 80045, USA

* Correspondence: yiqun.shellman@cuanschutz.edu; Tel.: +1-(303)-724-4034

Table S1: Full Canonical TBM Results Table. Experimentally validated TBMs are bolded and underlined.

Protein	Uniprot Code	Motif Seq	Starting position	Score	References
3BP2	P78314	**RSPPDGQS**	**415**	**0.80**	[1]
MCL1	Q07820	RNAVIGLN	6	0.45	
		RREIGGGE	45	0.35	
		REIGGGEA	46	0.65	
		**RPPPIGAE**	**78**	**0.69**	[1, 2]
		REQATGAK	187	0.48	
		RRVGDGVQ	214	0.89	
NUMA1	Q14980	RAGRKGLE	761	0.34	
		RLQQLGEA	770	0.34	
		RAEELGQE	1321	0.37	
		**RTQPDGTS**	**1743**	**0.79**	[1, 3]
		RRSQAGVS	1885	0.30	
		RDRHEGRK	2017	0.46	
AXIN1	O15169	**RPPVPGEE**	**22**	**0.38**	[1, 4]
		RRYSEGRE	284	0.49	
		REFRYGSW	290	0.32	
		RMEEEGED	417	0.54	
		RCVDMGCA	450	0.32	
		RSPDSGHV	492	0.32	
		RHRRTGHG	645	0.30	
		RRTGHGSS	647	0.61	
		RKVGGGSA	770	0.61	

		RTLVRGRA	797	0.31	
		RAVTLGQF	803	0.31	
TERF1	P54274	**RGCADGRD**	**13**	**0.78**	[1, 3]
		RAFRDGRS	88	0.45	
		RKYGEGNW	396	0.76	
FAT4	**Q6V0I7**	RGNEAGRF	178	0.29	
		RLQDEGTP	288	0.48	
		RLFTIGRH	1679	0.30	
		RAEDGGGQ	1917	0.31	
		RDDDRGSN	2285	0.33	
		RVFFAGFS	3698	0.16	
		RVTVGGIR	4106	0.29	
		RQSLRGAM	4230	0.32	
		RCVPPGDC	4423	0.49	
		**RKQPEGNP**	**4572**	**0.64**	[1]
		RHSPLGFA	4653	0.49	
		**RNPADGIP**	**4827**	**0.76**	[1]
DISC1	Q9NRI5	RARQCGLD	82	0.48	
		RVRAAGSL	170	0.46	
		**RGEAEGCP**	**223**	**0.65**	[1]
		REGLEGLL	618	0.47	
BABA1	Q9NWV8	**RSNPEGAE**	**28**	**0.70**	[1]
		**RSEGEGEA**	**48**	**0.82**	[1]
LKB1	Q15831	**RAKLIGKY**	**42**	**0.44**	[5]
		**RRIPNGEA**	**86**	**0.50**	[5]
AMPK	P54619	N/A	N/A	N/A	[5]

Table S2: Normalized score matrix for canonical and unconventional TBMs

	**Amino acid position within the peptide**							
**Amino acid**	**1**	**2**	**3**	**4**	**5**	**6**	**7**	**8**
**P**	-0.12953	0.014145	0.026554	0.047979	-0.12953	-0.12953	-0.12953	0.012124
**G**	-0.12953	0.013083	0.006632	0.175959	-0.12953	0.259067	0.008238	0.008238
**A**	-0.12953	0.011036	0.029326	0.023212	-0.12953	-0.12953	0.018679	0.017461
**V**	-0.12953	0.007694	0.009456	-0.12953	0.017876	-0.12953	0.007902	0.009689
**L**	-0.12953	0.018601	0.00671	-0.12953	-0.12953	-0.12953	0.00557	0.006606
**I**	-0.12953	0.007953	0.005104	-0.12953	0.009845	-0.12953	0.010363	0.007539
**M**	-0.12953	0.015699	0.011813	-0.12953	-0.12953	-0.12953	0.013523	0.008731
**C**	-0.12953	0.009948	0.010207	0.011917	0.009352	-0.12953	0.028031	0.012461
**S**	-0.12953	0.016451	0.017824	-0.12953	-0.12953	-0.12953	0.015466	0.012461

**T**	-0.12953	0.019119	0.009974	-0.12953	-0.12953	-0.12953	0.014223	0.007798
**R**	0.259067	0.005751	0.014508	-0.12953	-0.12953	-0.12953	0.007798	0.002642
**K**	-0.12953	0.005052	0.011166	-0.12953	-0.12953	-0.12953	0.007979	0.002332
**H**	-0.12953	0.008731	0.006865	-0.12953	-0.12953	-0.12953	0.012539	0.008083
**D**	-0.12953	0.019119	0.028705	-0.12953	0.161813	-0.12953	0.02114	0.048497
**E**	-0.12953	0.029456	0.027073	-0.12953	0.032358	-0.12953	0.030518	0.05456
**N**	-0.12953	0.006606	0.007772	-0.12953	-0.12953	-0.12953	0.013731	0.011788
**Q**	-0.12953	0.012409	0.01513	-0.12953	0.015337	-0.12953	0.019767	0.00671
**W**	-0.12953	0.011788	0.008679	-0.12953	-0.12953	-0.12953	0.007642	0.004741
**F**	-0.12953	0.011399	-0.12953	-0.12953	-0.12953	-0.12953	0.008834	0.007047
**Y**	-0.12953	0.015052	0.005596	-0.12953	0.012487	-0.12953	0.007098	0.009482

Table S3: Normalized score matrix for extended TBMs

	Amino acid position within the peptide					
**Amino acid**	**R1**	**G-2**	**G-1**	**G**	**G+1**	**G+2**
**P**	-0.13774	0.051019	-0.13774	-0.13774	-0.13774	0.012893
**G**	-0.13774	0.187107	-0.13774	0.275482	0.00876	0.00876
**A**	-0.13774	0.024683	-0.13774	-0.13774	0.019862	0.018567
**V**	-0.13774	-0.13774	0.019008	-0.13774	0.008402	0.010303
**L**	-0.13774	-0.13774	-0.13774	-0.13774	0.005923	0.007025
**I**	-0.13774	-0.13774	0.010468	-0.13774	0.011019	0.008017
**M**	-0.13774	-0.13774	-0.13774	-0.13774	0.01438	0.009284
**C**	-0.13774	0.012672	0.009945	-0.13774	0.029807	0.013251
**S**	-0.13774	-0.13774	-0.13774	-0.13774	0.016446	0.013251
**T**	-0.13774	-0.13774	-0.13774	-0.13774	0.015124	0.008292
**R**	0.275482	-0.13774	-0.13774	-0.13774	0.008292	0.00281
**K**	-0.13774	-0.13774	-0.13774	-0.13774	0.008485	0.002479
**H**	-0.13774	-0.13774	-0.13774	-0.13774	0.013333	0.008595
**D**	-0.13774	-0.13774	0.172066	-0.13774	0.022479	0.05157
**E**	-0.13774	-0.13774	0.034408	-0.13774	0.032452	0.058017
**N**	-0.13774	-0.13774	-0.13774	-0.13774	0.014601	0.012534
**Q**	-0.13774	-0.13774	0.016309	-0.13774	0.021019	0.007135
**W**	-0.13774	-0.13774	-0.13774	-0.13774	0.008127	0.005041
**F**	-0.13774	-0.13774	-0.13774	-0.13774	0.009394	0.007493
**Y**	-0.13774	-0.13774	0.013278	-0.13774	0.007548	0.010083

References for supporting information

1. Guettler, S., et al., *Structural basis and sequence rules for substrate recognition by tankyrase explain the basis for cherubism disease.* Cell, 2011. **147**(6): p. 1340-1354.
2. Bae, J., J.R. Donigian, and A.J.W. Hsueh, *Tankyrase 1 interacts with Mcl-1 proteins and inhibits their regulation of apoptosis.* Journal of Biological Chemistry, 2003. **278**(7): p. 5195-5204.
3. Sbodio, J.I. and N.-W. Chi, *Identification of a Tankyrase-binding Motif Shared by IRAP, TAB182, and Human TRF1 but Not Mouse TRF1 NuMA CONTAINS THIS RXXPDG MOTIF AND IS A NOVEL TANKYRASE PARTNER**. Journal of Biological Chemistry, 2002. **277**: p. 31887-31892.
4. Huang, S.M.A., et al., *Tankyrase inhibition stabilizes axin and antagonizes Wnt signalling.* Nature, 2009. **461**(7264): p. 614-620.
5. Li, N., et al., *Tankyrase disrupts metabolic homeostasis and promotes tumorigenesis by inhibiting LKB1-AMPK signalling.* Nature Communications, 2019. **10**(1).
